# spconfShiny: An R Shiny application for calculating the spatial scale of smoothing splines for point data

**DOI:** 10.1371/journal.pone.0311440

**Published:** 2024-10-04

**Authors:** Maddie J. Rainey, Kayleigh P. Keller

**Affiliations:** Department of Statistics, Colorado State University, Fort Collins, CO, United States of America; Villanova University, UNITED STATES OF AMERICA

## Abstract

Epidemiological analyses of environmental exposures often benefit from including spatial splines in models to account for confounding by spatial location. Understanding how the number of splines relates to physical spatial differences is not always intuitive and can be context-dependent. To address this, we developed a R Shiny application, spconfShiny, that provides a user-friendly platform to calculate an effective bandwidth metric that quantifies the relationship between spatial splines and the range of implied spatial smoothing. spconfShiny can be accessed at https://g2aging.shinyapps.io/spconfShiny/. We illustrate the procedure to compute the effective bandwidth and demonstrate its use for different numbers of spatial splines across England, India, Ireland, Northern Ireland, and the United States. Using spconfShiny, we show the effective bandwidth increases with the size of the region and decreases with the number of splines. Including 10 splines on a 10km grid corresponds to effective bandwidths of 92.2km in Ireland and 927.7km in the United States.

## Introduction

In studies that use regression models to estimate relationships between spatially-varying variables, such as air pollution concentrations or temperature and health outcomes, spatial confounding should be accounted for in the model [1]. Spatial confounding is defined as the presence of any unmeasured, spatially-varying factor that impacts a spatially-varying response variable when the main predictor is also spatially-varying. In epidemiological contexts, a common way to account for this confounding is to include adjustment for space via spatial splines [2–4]. Several two-step approaches have been introduced that incorporate splines in differing models and also have different approaches for choosing the number of splines to include in the models [5–8]. However, the relationship between the amount of spatial smoothing with a particular number of splines and the corresponding geographic distance is context-dependent. Generally, as additional splines are added to a model, finer spatial details can be modeled. But the size and shape of the geographic region can also impact the magnitude of the corresponding smoothing. A practical procedure is needed for interpreting the number of splines included in a spatial model in terms of spatial distances across different geographic regions.

R Shiny applications have become a beneficial tool to help researchers visualize and implement different spatial methodologies in their research [9–14]. For example, Salehi et. al. [9] created an application for the spatial visualization of COVID-19 data and Adin et. al [10] developed one for spatiotemporal disease mapping. Figueira et al. [11] developed an application, BAYSPINS, that implements a Bayesian approach for species distribution models, creating a tool for researchers who are less experienced with those types of models or researchers who want a quick way to implement them. In other contexts, Aparicio et. al. [12] developed the Mr.Bean app to visualize spatial information from agricultural field trials, Silva et. al. [13] developed the movedesign app for animal movement studies, and Johnson et. al. [14] developed an application, MBGapp, aimed at teaching geostatistical analyses to researchers that do not have much statistical training.

To aid in the interpretation of spatial smoothing for point-level data, we present an R Shiny application called spconfShiny, that calculates the spatial distance corresponding to a chosen number of splines for a particular set of spatial locations. spconfShiny implements a modification, described below, of a procedure first developed by Keller and Szpiro [7] for an effective bandwidth statistic. The core method is implemented in an accompanying R package, spconf [15]. Together, the package and application provide a user-friendly platform for researchers to calculate spatial scales for smoothing data from a custom set of geographic locations.

## Methods

### Statistical framework

The motivating context for this work is an epidemiological analysis of the association between a health outcome, *y*_*i*_(***s***_*i*_), and a spatially-varying exposure, *x*_*i*_(***s***_*i*_), for each individual with corresponding location, ***s***_*i*_. We assume that there are other measured covariates, $w_{i}\in R^{p}$. Unmeasured spatial confounding is a concern, so *J* spatial splines, which we denote *h*_*j*_(***s***_*i*_) for *j* = 1, …, *J*, are included in the model [5, 7]. A generalized linear model for estimating health effect associations in this context is:

$$\begin{array}{c} g\left(E\left[y_{i}\left(s_{i}\right)\right]\right)=f\left(x_{i}\left(s_{i}\right),\beta \right)+w_{i}^{T}\gamma +\sum_{j=1}^{J}\alpha_{j}h_{j}\left(s_{i}\right) \end{array}$$ (1)

where E[*y*_*i*_(***s***_*i*_)] is the mean of the response, *g*(⋅) is a link function, $f(x_{i}\left(s_{i}\right),\beta )$ is an exposure-response function, $\gamma \in R^{p}$ are regression coefficients for the measured covariates, and $\alpha_{j}\in R$ are the regression coefficients of the splines. We choose *g*(⋅) to be the identity link, assuming that our response is continuous; however, other links may be assumed if the response is discrete.

Increasing the number of splines, *J*, included in Eq 1 allows for finer scale spatial adjustments in the model. However, larger values of *J* do not necessarily equate to lower bias in the exposure-response association estimate [7]. Decisions of how many splines to include should consider whether exposures are predicted or measured and the magnitude of non-spatial variation in exposure due to the possibility of over adjusting and nullifying the estimated association by adding too many splines [5, 7]. The choice of the number of splines to include in the model is beyond the scope of this work but is an active area of research.

The target of the inferential analysis in Eq 1 is to estimate the exposure-response relationships summarized by the parameter ***β***; however, the goal of this work is to provide an interpretation of the scale of the spatial splines *h*_1_(***s***_*i*_), …, *h*_*J*_(***s***_*i*_) so that the estimate for ***β*** can be interpreted more precisely.

### Effective bandwidth of spatial splines

The common choice of basis to create spatial splines is the thin-plate regression spline (TPRS) basis [16], which can be calculated in R via the mgcv package [17]. For unpenalized splines, the degrees of freedom (df) of a basis is equal to the number of splines, represented by *J* in Eq 1. To interpret the choice of df for the TPRS basis, we propose an effective bandwidth, which we denote $\hat{k}$, using an approach adapted from a procedure developed by Keller and Szpiro [7]. We interpret the effective bandwidth as the approximate minimum radius of the area over which points are smoothed. In the context of Eq 1, we can also think of the effective bandwidth as, given a specific location, the minimum distance at which confounding is being adjusted. In an epidemiological context, the inclusion of spatial splines can be interpreted as a means for adjusting for confounding by location over a range given by the effective bandwidth. Smaller values of $\hat{k}$ mean that fewer locations are averaged across and thus finer-scale spatial details are adjusted for in Eq 1.

**Fig 1:**
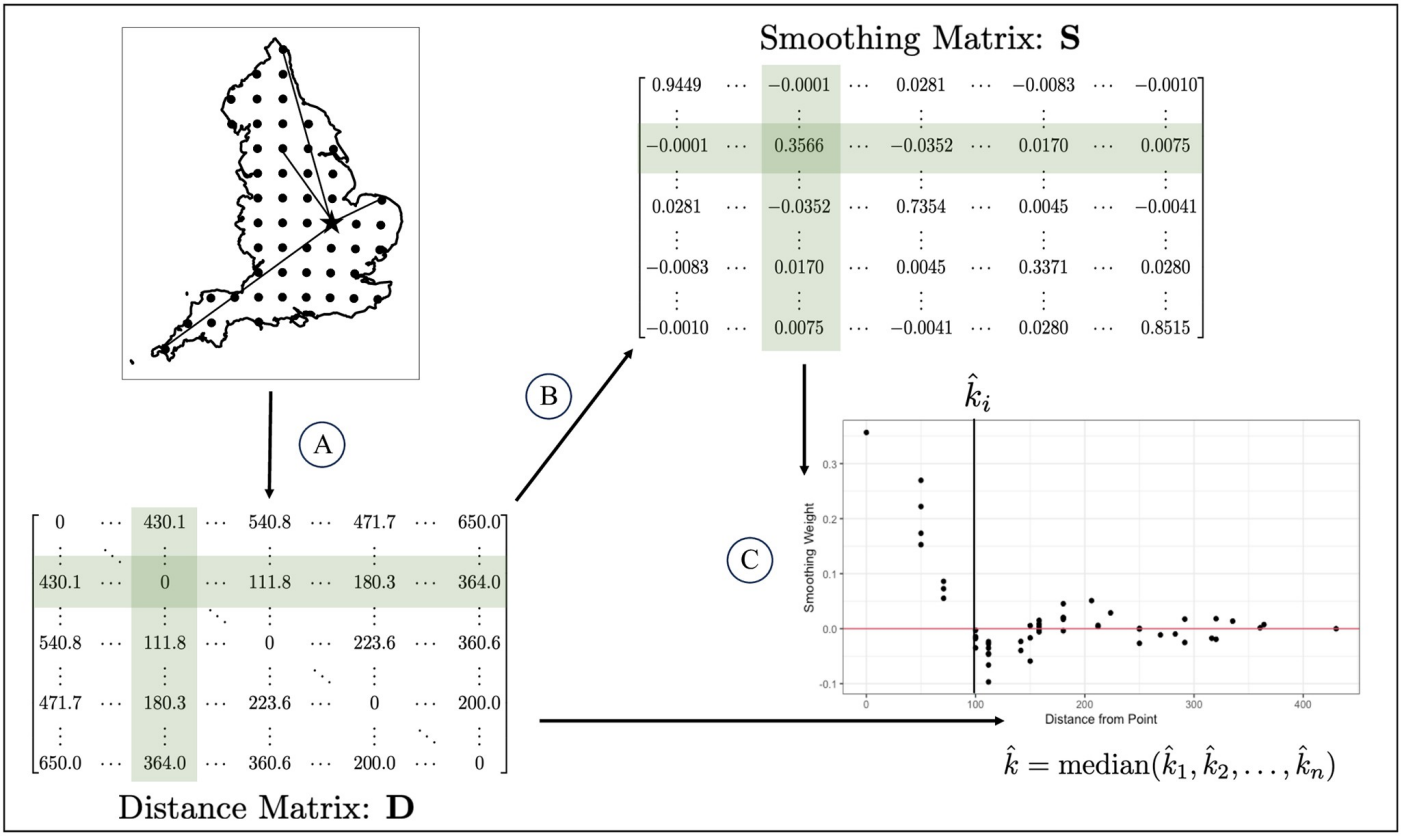
Visual representation of the process of computing the effective bandwidth. Step A computes the distance matrix, **D**, of the 50km grid across England. A smoothing matrix, **S**, is computed from the information in **D** via a TPRS basis in step B. The highlighted rows and columns correspond to the distance and smoothing values for the starred point. For each point in the grid, the smoothing weights are ordered by distance and the smallest distance with a negative smoothing weight, ${\hat{k}}_{i}$, is obtained and visually represented in step C. Finally, the effective bandwidth is computed by taking the median of the ${\hat{k}}_{i}$.

The process of computing the effective bandwidth is illustrated in Fig 1 and in Algorithm 1. To determine $\hat{k}$, we first calculate a value ${\hat{k}}_{i}$ for each location *i* (or a random subset of locations). For a set of points $S=\{s_{1},\ldots ,s_{n}\}$, we first obtain the Euclidean distance matrix, $D\in R^{n\times n}$ between the given coordinates (step A in Fig 1). A TPRS basis, $H\in R^{n\times (df+1)}$, is computed based on ***D*** and is used to compute the smoothing matrix, $S=H{\left(H^{T}H\right)}^{-1}H^{T}\in R^{n\times n}$ (step B in Fig 1). For each column ***S***[, *i*], we order the values by the corresponding distances to all other points and find the distance at which the values from ***S***[, *i*] first cross zero (step C in Fig 1). The median of these distances, ${\hat{k}}_{i}$, is what determines $\hat{k}$.

**Algorithm 1** Computational algorithm for computing the effective bandwidth

**Require**: $S\in R^{n\times 2}$

 Initialize *k*_*vec*_[*n*]

 Compute TPRS basis, $H\in R^{n\times (df+1)}$

D=distance(S,S)

$S=H{\left(H^{T}H\right)}^{-1}H^{T}$

 **for** *i* ∈ 1 : *n* **do**

  order ***S***[, *i*] by increasing ***D***[, *i*]

  *k*_*vec*_[*i*] = *min*(***D***[, *i*]) where ***S***[, *i*] < 0

 **end for**

 *k* = median(*k*_*vec*_)

 **return** *k*

#### Keller and Szpiro’s effective bandwidth

The procedure we developed to compute the effective bandwidth contrasts the methodology developed by Keller and Szpiro [7] in how the relationships between the distances and the smoothing weights are used. In place of our Step C (Fig 1), Keller and Szpiro [7] fit a loess curve to the smoothing weights (***S***[, *i*]) as a function of distance, which also requires selecting a span value that controls the proportion of points included in the smoothing. They then predict smoothing weights for a set sequence of new distances and define the effective bandwidth as the distance at which the median predicted smoothing weights first cross zero. Our proposed methodology orders the empirical smoothing weights by distance and finds the smallest distance that has a negative smoothing weight, effectively finding where the points first cross the x-axis when plotting the smoothing weights by distance. The median of the selected distances determines the effective bandwidth. Compared to the original approach of Keller and Szpiro [7], our approach does not require the user to input the span for the smoothing calculations. This makes our approach faster and more user-friendly for differing geographic regions. However, we expect there to be a difference between the two computations. Using a loess curve to compute the effective bandwidth averages over a neighborhood of distances, creating an average radius for the area that is smoothed. In comparison, our proposed method takes the first point that below zero, not considering any other points, creating a minimum radius for the same area.

## Computing the effective bandwidth in spconfShiny

spconfShiny is an interactive Shiny web application based on the spconf package in the R language, updated with our adaptation of the effective bandwidth [15]. We have integrated the modified effective bandwidth into spconf, which also retains functions for computing the version of the bandwidth measure proposed by Keller and Szpiro [7].

### Coordinate input options

In spconfShiny, we provide three different options to obtain spatial coordinates to compute the effective bandwidth:

- Create gridded coordinates in the application
- Select a set of preloaded coordinates
- Upload coordinates from a user file.

To create gridded coordinates, the length and the width of the grid must be entered and the user must select the distance between points (grid increment size). The preloaded coordinates in the application currently include the countries of England, India, Ireland, Northern Ireland, and the contiguous United States with grid sizes of 10km, 50km, 10km, 1km, and 50km, respectively. The user uploaded coordinates should be in .csv format, and the user must indicate the names of the columns that include the spatial coordinates.

### Effective bandwidth options

The maximum number of splines must be selected in order to compute the effective bandwidth. The application offers the choice of 10, 25, 100, 300, or 500 splines. However, the number of splines may not exceed the number of coordinates included in the computations. The calculations slow as the number of coordinates increases; therefore, the application offers the option to subsample the coordinates to 1000, 2000, or 5000 locations to reduce computation time. If the number of coordinates in the computation is smaller than the selected number of points to subsample, all coordinates will be used.

### Computing the effective bandwidth

To compute the effective bandwidth, the application first computes unpenalized TPRS on the coordinates via the computeTPRS() function from the spconf package [15] with the chosen maximum number of splines. The computeTPRS() function relies on the mgcv and stats packages [17, 18]. Then, for each df between 3 and the maximum df, the effective bandwidth is computed using the compute_effective_range() function from the spconf package [15], which implements Algorithm 1 and relies on functions from the stats and flexclust packages [18, 19]. The application provides the output in both a tabular and graphical form, selected by switching tabs. A plot of the coordinates is also displayed in a third tab. The tabular results are available to download in .csv format.

### Shiny implementation

The spconfShiny application (deployed at https://g2aging.shinyapps.io/spconfShiny/) is implemented by the Shiny package [20] and the Shiny implementation also uses the shinyjs, shinyWidgets, and bslib packages [21–23] with plots created by ggplot2 [24]. Additional parallelization of the smoothing curve estimation is done by the parallel package [18].

## Demonstration of spconfShiny across different geographic regions

To demonstrate the utility of the application, we compared spatial bases created across England, India, Ireland, Northern Ireland, and the contiguous United States, which represent a range of different geographic sizes and are locations of current studies investigating the impacts of aging on cognition [25]. We obtained shapefiles for these countries from Natural Earth [26]. For each country, we created grids using Transverse Mercator projected coordinate system for England (1km, 10km, and 25km), Ireland (1km, 10km, and 25km), and Northern Ireland (1km and 10km) and Lambert Conformal Conic projected coordinate system for India (10km, 25km, and 50km) and the United States (10km, 25km, and 50km).

**Fig 2:**
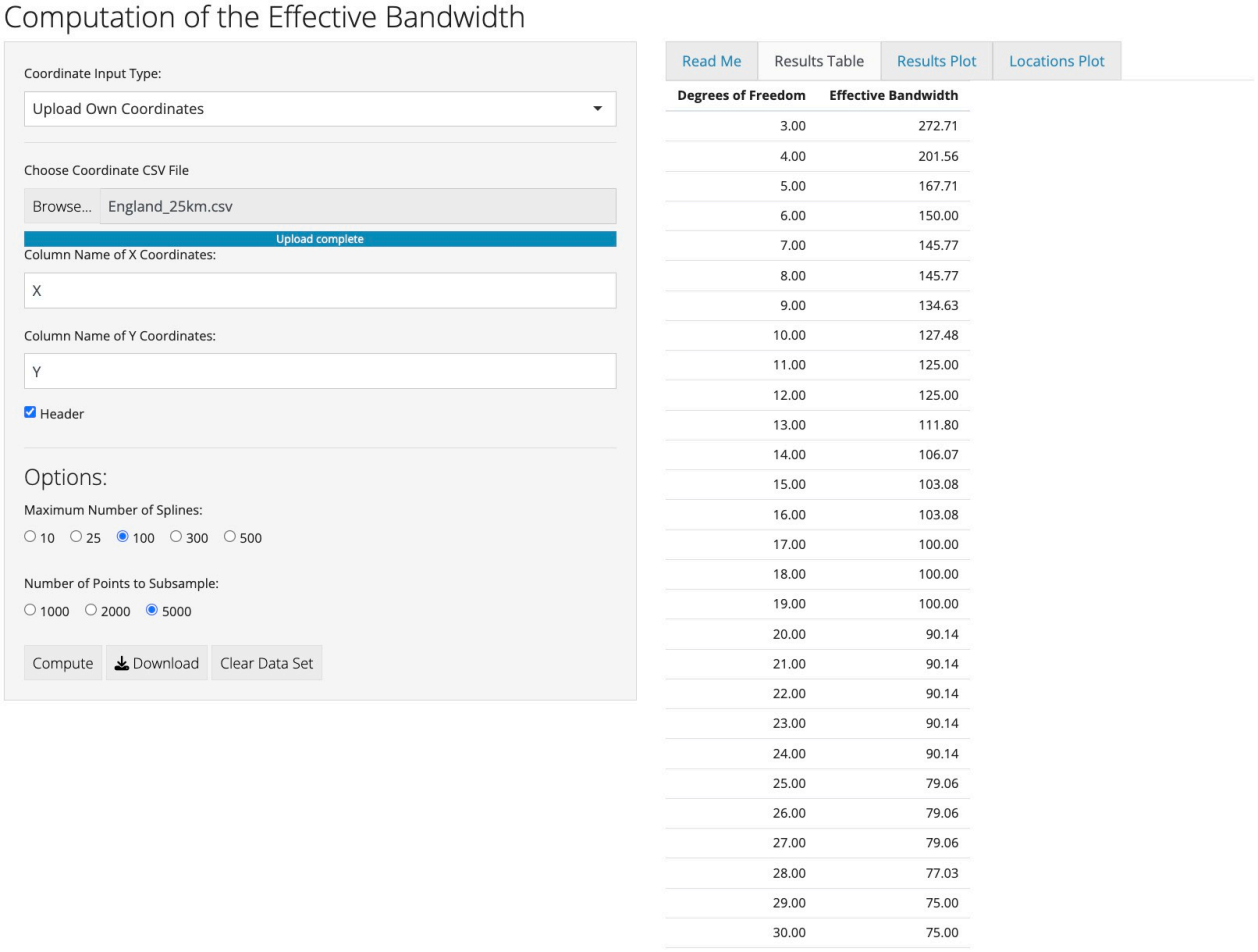
spconfShiny output for user inputted 25km grid across England.

Using England with a 25km grid as an example, we uploaded the coordinates in the ‘File Input:’ section of the application. We then selected to compute the effective bandwidth for 100 splines and used either all points in the dataset or sampled 5000, whichever was smaller. After clicking the compute button, we downloaded the table of effective bandwidths and summarized the results for 5, 10, 25, 100 df in Table 1. An image of the application is shown in Fig 2. We proceeded with the other countries and grid sizes similarly. For countries with grids that have more that 300 points, 300 df was also summarized in the table.

**Table 1:** Effective bandwidth estimates, interpretable in kilometer distances, for thin-plate regression splines evaluated on different grid sizes across five countries. df indicates degrees of freedom.

Country	Grid Size	5 df	10 df	25 df	100 df	300 df
England	1km	156.4	124.3	77.2	37.6	21.0
	10km	160.3	125.3	80.0	40.0	28.3
	25km	167.7	127.5	79.1	50.0	–
India	10km	869.8	628.1	386.4	190.0	106.3
	25km	822.3	636.4	391.3	195.3	111.8
	50km	838.2	650.0	400.0	200.0	141.4
Ireland	1km	125.0	88.1	56.3	27.9	15.6
	10km	130.0	92.2	58.3	30.0	20.0
	25km	127.5	100.0	70.7	35.4	–
Northern Ireland	1km	53.5	39.5	25.0	12.6	7.1
	10km	56.6	41.2	28.3	14.1	–
United States	10km	1178.3.0	930.0	593.0	294.1	162.8
	25km	1153.9	927.7	594.2	300.0	167.7
	50km	1192.7	948.7	602.1	304.1	180.3

### Comparison of the effective bandwidth

**Fig 3:**
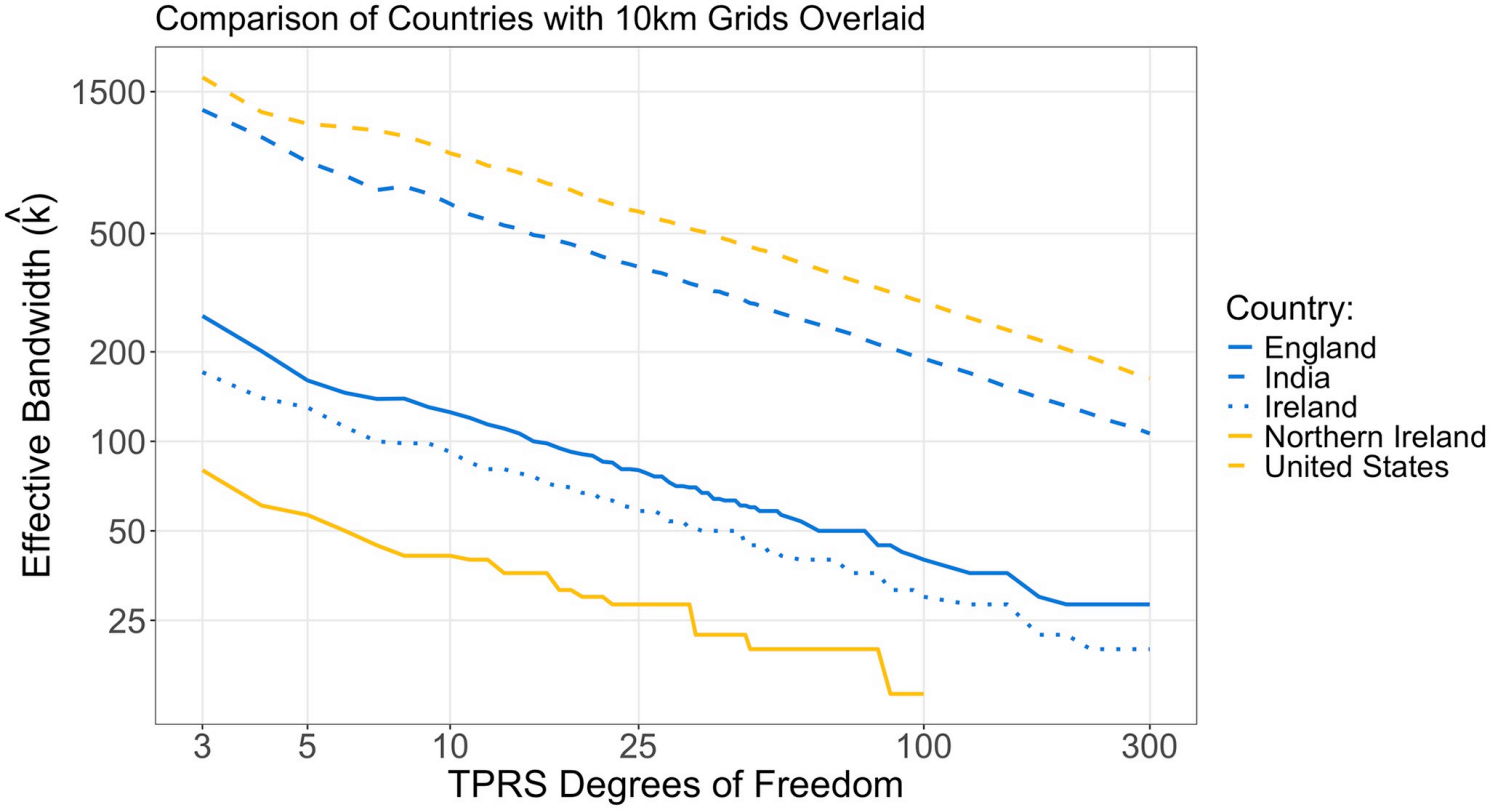
Comparison of the effective bandwidth computed for TPRS created on the 10km grid across England, India, Ireland, Northern Ireland, and the United States.

Among the five countries that we compared, the least number of points that was considered was 115 for the 25km grid across Ireland and the most points considered was 130,382 for the 1km grid across England (Table 2). The smallest area that we compared was Northern Ireland, and the largest area that we compared was The United States (boundary height and width of 436km and 117km, and 2890km and 4610km, respectively: Table 2). Comparing the same df for the different countries, on the same grid size, $\hat{k}$ is smaller for smaller countries compared to larger countries: $\hat{k}$ of 41.2km, 92.2km, 125.3km, 628.1km, and 930.0km, for Northern Ireland, Ireland, England, India and the United States, respectively, for a TPRS basis with 10df on a 10km grid (Table 1, Fig 3).

**Table 2:** Characteristics of grids used to compute the effective bandwidths.

Country	Boundary	Boundary	Points in Grid			
	Width (km)	Height (km)	1km	10km	25km	50km
England	567	646	130,382	1,302	210	–
India	2,840	3,090	–	32,558	5,217	1,300
Ireland	303	436	69,431	701	115	–
Northern Ireland	117	141	14,250	141	–	–
The United States	4,610	2,890	–	79,230	12,665	3,173

Comparing different grid sizes for the same country, for the same df, the grid size does not have a meaningful influence on $\hat{k}$ ($\hat{k}$ of 628.1km, 636.4km, and 650.0km, for a TPRS basis of India with 10df with grid sizes of 10km, 25km, and 50km, respectively: Table 1). However, a user must still have reasonably fine resolution across the area as there must be more points than df included in the model.

### Using the effective bandwidth in epidemiological studies

Ideally, the choice of the effective bandwidth, or number of splines included in Eq 1, should be made before completing an analysis. When selecting an effective bandwidth, researchers should consider the relationship between the effective bandwidth, the complexity of the model, and the amount of spatial smoothing induced. Smaller effective bandwidths require more spatial splines to be included in the model, increasing the model complexity due to increasing the number of coefficients needed to be estimated. However, as stated previously, including more splines does not always equate to more accurate exposure-response association estimates [7]. The number of locations also affects the effective bandwidth since the maximum number of splines that can be created is equivalent to the number of locations. Thus, some effective bandwidths may not be attainable due to the lack of spatial information in the data. spconfShiny can facilitate comparisons between similar sized countries for researchers who want to ensure the same amount of spatial smoothing. This can either be done by selecting an effective bandwidth, and determining the df needed for each country to spatially smooth at that range; or selecting the proportion of area of each country to smooth over, determining the effective bandwidth necessary for each country to achieve that proportion, and then determining the df needed for that effective bandwidth.

Suppose we wanted to compare a minimum smoothing radius of 100km in England and Ireland with a 10km grid. Using the Shiny application, we determine that we will need to include 7 df in the analysis for Ireland and 15 df in the analysis for England. However, if we want to smooth over the same proportion of area, for example 0.1 (i.e., 10% of the region), we need effective bandwidths of approximately 64km for England and 52km for Ireland, corresponding to including 36 df and 32 df in the analysis, respectively. Similarly, suppose we wanted to compare India and the United States with a 50km grid and want a minimum smoothing radius of 500km, we need 16 df included in the analysis for India and 36 df included in the analysis for the United States.

### Comparison with alternative approaches

Finally, we provide two sensitivity analyses: a comparison of our proposed variant of the effective bandwidth with the original method of Keller and Szpiro [7], and a comparison of our proposed approach using TPRS splines and using Duchon splines [27]. First, we compare the variant of the effective bandwidth with the original method for England with a 10km grid and the United States with a 50km grid using bases containing 5, 10, 25, and 100 df. For comparison with our proposed approach, we applied Keller and Szpiro’s [7] method with spans of 0.1 and 0.5 (representing 10% and 50% of the data included in the loess curve smoothing). The original effective bandwidth produces larger values for the bandwidth than our variant (Table 3). This difference is to be expected since the proposed approach calculates minimum smoothing radius while the Keller and Szpiro [7] approach calculates an average smoothing radius. The difference between the two methods decreases as the number of df in the basis increases (Table 3). Although both methods provide an effective bandwidth estimate, it is important that researchers use the same method when comparing across contexts.

**Table 3:** Effective bandwidth estimates for England with a 10km grid and the United States with a 50km grid comparing the original method of computing the effective bandwidth and our proposed computation. span = 0.1 and span = 0.5 indicate the original method with stated spans for the loess computation. df indicates degrees of freedom.

Country	Method	5 df	10 df	25 df	100 df
England	span = 0.1	268.6	156.9	98.2	52.9
	span = 0.5	268.2	157.9	102.4	87.6
	new	160.3	125.3	80.0	40.0
United States	span = 0.1	1660.4	1120.0	682.0	334.9
	span = 0.5	1658.7	1126.8	708.8	581.3
	new	1192.7	948.7	602.1	304.1

We use the same countries and degrees of freedom to compare the choice of spatial basis. We compare the TPRS basis with low rank Duchon splines. Duchon splines are a broader class of spatial splines that encompasses thin plate splines [27]. For our comparison, we used the low-rank form of Duchon splines, implemented in the mgcv package [17] The Duchon splines are used as an input for the compute_effective_range() function from the spconf package, which provided the calculation of the effective bandwidth underlying the spconfShiny package [15]. The Duchon splines produce smaller effective bandwidths than the TPRS for smaller degrees of freedom, eventually converging to the same bandwidth as the df increases (Table 4). While either set of splines could be used in practice, we implement only TPRS in spconfShiny due to their widepspread use in spatial analyses.

**Table 4:** Effective bandwidth estimates for England with a 10km grid comparing using TPRS or low rank Duchon splines to compute the spatial basis. df indicates degrees of freedom.

Country	Basis	5 df	10 df	25 df	100 df
England	TPRS	160.3	125.3	80.0	40.0
	Duchon	122.1	100.0	70.0	40.0
United States	TPRS	1192.7	948.7	602.1	304.1
	Duchon	930.1	743.3	522.0	304.1

## Conclusion

spconfShiny is a Shiny application that creates a user-friendly interface for the computation of the effective bandwidth for spatial splines. The effective bandwidth quantifies the amount of spatial smoothing induced in a model by including a given number of spatial splines in a model. Using the effective bandwidth, we can compare the impact of spatial smoothing across different geographic regions for differences in size and shape. As seen in our demonstration of spconfShiny, when creating models that will be applied to studies in different sized regions, different degrees of freedom should be used to model the same level of spatial detail and the smaller region will require including fewer splines compared to the larger region.
